# A standardized herbal extract mitigates tumor inflammation and augments chemotherapy effect of docetaxel in prostate cancer

**DOI:** 10.1038/s41598-017-15934-0

**Published:** 2017-11-15

**Authors:** Chin-Hsien Tsai, Sheue-Fen Tzeng, Shih-Chuan Hsieh, Yu-Chih Yang, Yi-Wen Hsiao, Mong-Hsun Tsai, Pei-Wen Hsiao

**Affiliations:** 1 Agricultural Biotechnology Research Center, Academia Sinica, Taipei, Taiwan; 2 Graduate Institute of Life Sciences, National Defense Medical Center, Taipei, Taiwan; 3 Bioinformatics and Biostatistics Core, Center of Genomic Medicine, National Taiwan University, Taipei, Taiwan; 4 Institute of Biotechnology, National Taiwan University, Taipei, Taiwan

## Abstract

Activation of the NFκB pathway is often associated with advanced cancer and has thus been regarded as a rational therapeutic target. *Wedelia chinensis* is rich in luteolin, apigenin, and wedelolactone that act synergistically to suppress androgen receptor activity in prostate cancer. Interestingly, our evaluation of a standardized *Wedelia chinensis* herbal extract (WCE) concluded its efficacy on hormone-refractory prostate cancer through systemic mechanisms. Oral administration of WCE significantly attenuated tumor growth and metastasis in orthotopic PC-3 and DU145 xenografts. Genome-wide transcriptome analysis of these tumors revealed that WCE suppressed the expression of IKKα/β phosphorylation and downstream cytokines/chemokines, e.g., IL6, CXCL1, and CXCL8. Through restraining the cytokines expression, WCE reduced tumor-elicited infiltration of myeloid-derived suppressor cells (MDSCs), tumor-associated macrophages (TAMs) and endothelial cells into the tumors, therefore inhibiting angiogenesis, tumor growth, and metastasis. In MDSCs, WCE also reduced STAT3 activation, downregulated S100A8 expression and prevented their expansion. Use of WCE in combination with docetaxel significantly suppressed docetaxel-induced NFκB activation, boosted the therapeutic effect and reduced the systemic toxicity caused by docetaxel monotherapy. These data suggest that a standardized preparation of *Wedelia chinensis* extract improved prostate cancer therapy through immunomodulation and has potential application as an adjuvant agent for castration-resistant prostate cancer.

## Introduction

Although current hormonal and chemotherapeutic approaches improve survival in patients with prostate cancer (PCa), relapse typically occurs after 1–2 years and often results in lethality^1^. PCa is usually complicated by tissue inflammation which is closely associated with higher grade PCa^2^. On the other hand, long-term and regular use (more than 30 tablets a month for longer than 5 years) of non-steroid anti-inflammatory drugs (NSAIDs), such as aspirin has been associated with modestly lower risk of PCa incidence^3^. It is not inconceivable that the inflammatory tumor microenvironment may contribute to the therapeutic progression and comprise therapeutic targets. Oncogenes, cytokines, chemokines and their receptors orchestrate the inflammatory tumor microenvironment and continuous infiltration of immune cells aids progression to more aggressive stages. Inflammation-derived cytokines are also involved in promoting the oncogenesis and progression of PCa; for example, elevated serum IL6 levels correlate with poor survival and promote castration-resistance in PCa, while IL8 (CXCL8) promotes AR transcriptional activity^4,5^. Inflammatory cytokines and chemokines not only mediate inflammatory reactions but also participate in the tumor growth, castration-resistance, invasion, and metastasis of PCa cells^6–8^. Among these, CXCL1, CXCL5, and CXCL8 have been described to stimulate PCa cell proliferation, cell survival, migration, invasion, and angiogenesis, thus promoting tumor growth as well as metastasis^9,10^.

Although docetaxel-based therapy benefits the overall survival of patients with castration-resistant PCa (CRPC), eventually all CRPCs progress to docetaxel-resistant disease^11^. Since docetaxel is the first known agent to extend survival in men with CRPC, numerous phase III clinical trials have investigated the use of novel agents either as adjuvant or neoadjuvant with docetaxel. Application of nutraceuticals, such as curcumin, to supplement conventional therapy was found to be well tolerated and accepted by patients in clinical trials^12^. However, these results failed to meet the primary endpoint of improving overall survival^13^. Clinical studies showed that docetaxel-treated patients exhibit increased CXCL8 expression, and upregulation of CXCL8 promotes the survival of PCa cells rendering docetaxel treatment less effective^14,15^. Therefore, inflammation in the cancer microenvironment also compromised treatment outcome of chemotherapy.

Herbal medicines may contain multiple active compounds that elicit synergistic actions by targeting either the same or different molecules in various pathways in PCa cells as well as other different cell types, improve the pharmacokinetics of drug compounds by regulating the enzymes and transporters involved in hepatic and intestinal metabolism, and overcome the drug resistance of cancer cells, or eliminate the adverse toxicity to enhance pharmacological efficacy of chemotherapeutic drugs. *Wedelia chinensis* is a medicinal herb with anti-PCa activity^16^. *In vivo* study showed that *Wedelia chinensis* extract inhibited the function of androgen receptor (AR) signaling and the tumor growth of AR-positive PCa without adverse toxicity as observed by body weight and physical activities^17^. Previously, we developed a standardized preparation of *Wedelia chinensis* extract (WCE) which ensures optimized effect against AR activity in PCa cells and equivalent efficacy and chemical profile in each lot^18^. In addition, we demonstrated that the context of herbal preparation improves the pharmacokinetic profile of the active compounds, wedelolactone, apigenin, and luteolin, by increasing their half-lives and therefore enhancing the tumor suppression effect compared to the formula of pure active compounds^18^. However, the effect of WCE on CRPC has not been characterized. Here, we further studied the *in vivo* effect of WCE on a CRPC model and demonstrated that among the three active components, wedelolactone simultaneously suppresses IKKα/β signaling in cancer cells and modulates the activity of tumor-induced myeloid cells. WCE as an anti-inflammatory therapy inhibited the growth of CRPC and also exhibited a beneficial effect when used in combination with docetaxel chemotherapy by intensifying the anti-tumor effect and reducing docetaxel-induced cytokine expression and toxicity.

## Results

### Effect of *Wedelia chinensis* extract on the cell growth of AR-positive and negative prostate cancer cells

**Figure 1:**
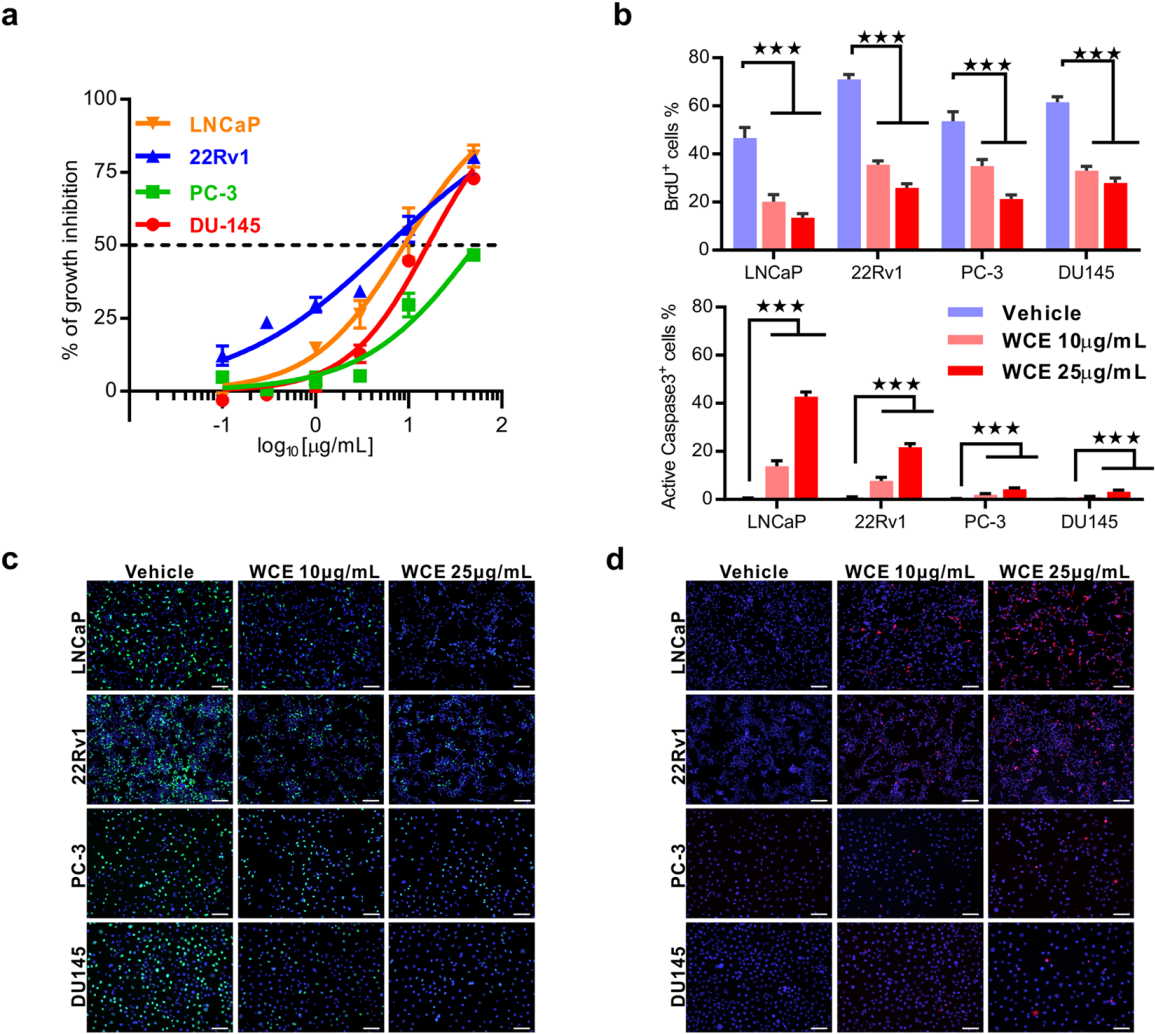
Effect of WCE on cell growth in different types of PCa cells. (**a**) The growth curves of 22Rv1, LNCaP, DU145 and PC-3 treated with different concentrations of WCE were measured using DNA content measurement and normalized to vehicle treatment. (**b**) The proliferation index and apoptosis rate were determined by BrdU incorporation assay and immunofluorescent staining for active caspase 3, respectively. (**c**,**d**) The representative images of anti-BrdU antibody staining (**c**) and anti-active caspase 3 antibody staining (**d**) in four PCa cell lines. Bar, 100 μm. Data are presented as means ± SEM. ***P ≤ 0.001. (t-test, two tail).

The active compounds in *Wedelia chinensis* extract (WCE), luteolin, apigenin, and wedelolactone are known to effectively suppress cell growth of AR-expressing PCa cells^16^. Resistance to hormonal therapy remains a major threat to PCa patients. Because luteolin and apigenin in WCE effectively inhibited the *in vitro* growth of hormone-refractory PCa cell lines, we were inspired to investigate the anticancer effects of WCE on hormone-refractory PCa. *In vitro*, WCE dose-dependently suppressed the growth of both AR-expressing PCa cells (LNCaP and 22Rv1) and hormone-refractory PCa cells (PC-3 and DU145) (Fig. 1a). Although exposure to WCE significantly impaired the cell proliferation in all these PCa cell lines as determined by BrdU incorporation assay, WCE had a more profound proapoptotic effect (caspase 3 activation) in 22Rv1 and LNCaP cells than in PC-3 and DU145; where the apoptotic cell populations were 25–45% *vs*. below 4–5%, respectively, in the presence of 25 μg/mL high-dose WCE (Fig. 1b–d). These data indicate that WCE may act as cytostatic agent in hormone-refractory PCa and therefore the *in vivo* response merited further exploration.

### WCE suppresses *in vivo* tumor growth and spontaneous metastasis of hormone-refectory PCa

**Figure 2:**
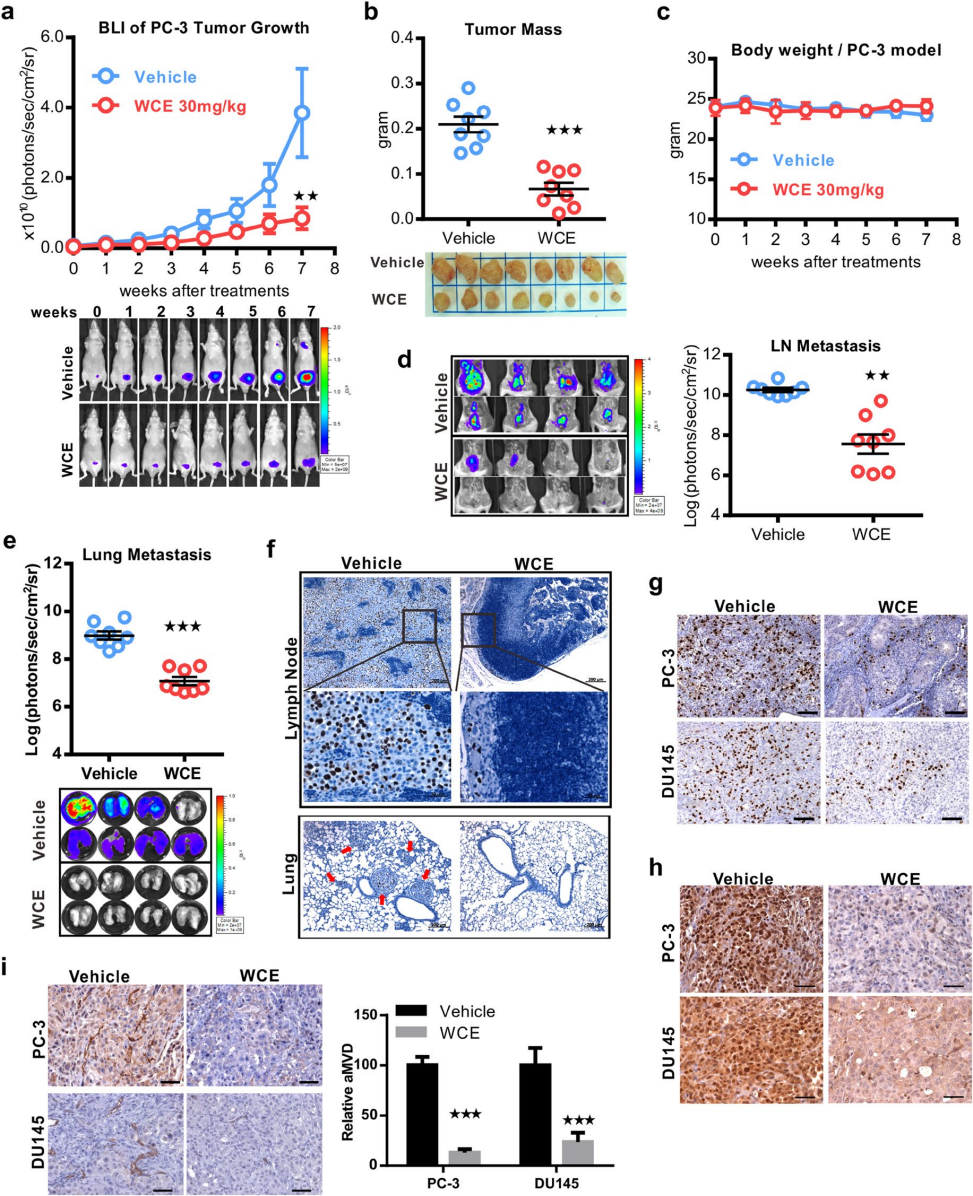
Therapeutic effect of WCE on an orthotopic xenograft model of hormone-refractory PCa. (**a**) Longitudinal bioluminescence images (BLI) of PC-3-Luc2 prostate tumors in each group were measured weekly and are presented as a growth curve (n = 8). Bottom, the longitudinal images of BLI in representative mice. (**b**) The tumor weight following 7-week treatment is represented as a column scatter plot. Horizontal lines, mean (n = 8); Bottom, photograph of representative primary tumors taken at the endpoint. (**c**) Mouse body weight was monitored weekly. (**d**,**e**) Effect of WCE on tumor metastasis. BLI of the host lung (**d**) and lumbar lymph nodes (**e**) were examined at the endpoint. Metastasis was quantified by BLI as photon flux per second, and the results are represented as a column scatter plot (n = 8). (**f**) Immunohistochemical staining of metastases in lumbar lymph nodes and lungs for Ki-67 to indicate proliferating PCa cells. Arrows indicate cancer cells. (**g**) Anti-BrdU antibody was applied to determine proliferative cells in tumors. Scale bar, 100 μm. (**h**) IHC staining of HIF1α in PC-3 and DU145 tumor tissues. Scale bar, 50 μm. (**i**) The neovasculature of tumors was determined by CD31 staining in PC-3 and DU145 tumor slices and quantified as relative positive area (area microvessel density, aMVD) from four random fields in one tumor slice, n = 3 (right). Scale bar, 50 μm. All values are represented as means ± SEM. **P ≤ 0.01; ***P ≤ 0.001 (t-test, two-tail).

A nude mouse model bearing orthotopic PC-3 or DU145 tumors were orally treated with 10 mg/kg/day WCE for 7 weeks, and the tumor growth and metastasis were evaluated by quantitative bioluminescence imaging (BLI). Although WCE induced cell apoptosis in AR-positive LNCaP and 22Rv1 cells more significantly than in AR-negative PC-3 and DU145 cells, unexpectedly, the results showed that WCE also significantly inhibited the tumor growth without affecting the body weight in both the mouse models (Fig. 2a–c and Supporting Information Fig. S1a–c). In parallel with the tumor growth, BLI analysis of lung and lymph nodes showed that WCE significantly repressed the metastasis of cancer cells compared to vehicle treatment (Fig. 2d and e, and Supporting Information Fig. S1d and e). Pathological analysis further confirmed that there were only a few proliferating PCa cells in the lymph nodes stained by human-specific Ki-67, and such PCa cells were hardly detected in the lung specimens of PC-3 tumor-bearing mice (Fig. 2f). Results of *in vivo* BrdU incorporation assay also showed that WCE treatment greatly impedes the PCa cell proliferation in the mouse model (Fig. 2g). Hypoxic stress response and associated angiogenesis are typical features observed in various tumors. To this end, HIF1α expression in the WCE-treated tumors was found far below the control-treated tumors (Fig. 2h). Accordingly, the tumor angiogenesis as detected by CD31 staining was significantly impaired by WCE treatment (Fig. 2i). At necropsy examination, we found that tumor-associated splenomegaly was ameliorated by WCE treatment, indicating the WCE-mediated improvement of disease symptoms may involve immune modulation (Supporting Information Fig. S1f). These results show that oral WCE treatment hampered the prostatic tumor development and has the potential to mediate the tumor microenvironment.

### WCE inhibits NFκB signaling and downregulates cytokines in cancer cells

**Figure 3:**
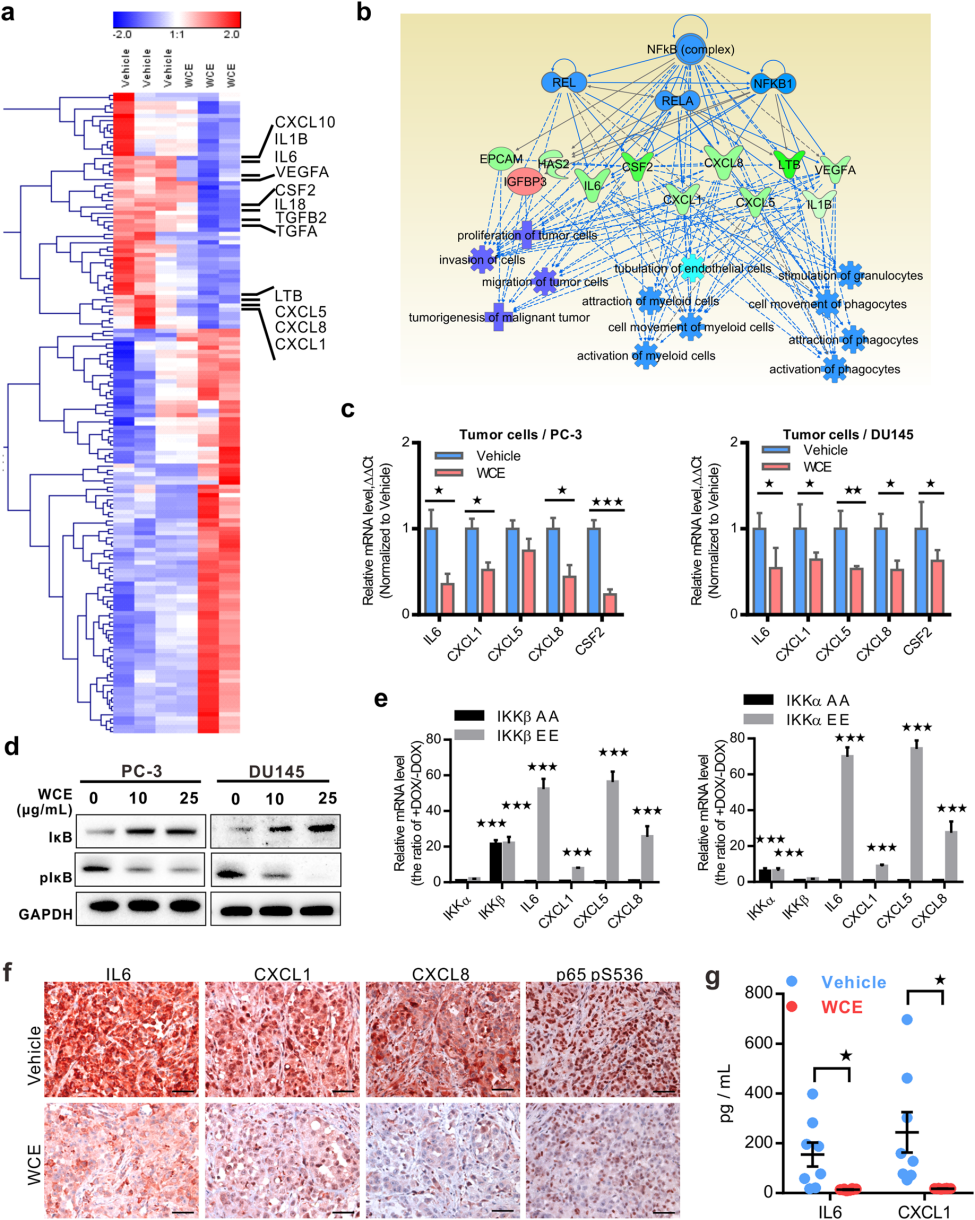
WCE-mediated transcriptome analysis in PCa tumor samples. (**a**) A heat map of WCE-regulated differential genes analyzed in RNA-seq. (**b**) The potential upstream regulators of WCE-mediated gene signatures were extracted from IPA. (**c**) The mRNA levels of proinflammation cytokine/chemokine genes in PC-3 (Left) or DU145 (Right) tumor tissues were analyzed by qRT-PCR. (**d**) IκB and pIκB in PC-3 and DU145 cells were determined in WCE treatment. (**e**) The mRNA level of IKKα, IKKβ, and WCE-regulating genes were examined in PC-3 cells transfected with constitutive active IKK-EE mutant or dominant negative IKK-AA mutant by qRT-PCR. The gene expression was induced by doxycycline (DOX, 1 μg/mL) and the value was normalized by + DOX/–DOX. (**f**) The expression of IL6, CXCL1, CXCL8, and p65 (pS536) in the primary PC-3 tumors was stained by IHC. Scale bar, 50 μm. (**g**) Plasma levels of IL6 and CXCL1 in PC-3 tumor-bearing mice were determined by ELISA after WCE or vehicle treatment (n = 8). All values are represented as means ± SEM. *P ≤ 0.05; **P ≤ 0.01; ***P ≤ 0.001 (t-test, two-tail).

To identify the mechanism of WCE for this anticancer action, vehicle-treated and WCE-treated PC-3 tumor tissues were analyzed for their differential genes using the RNA-seq approach (GSE99820). A group of cytokine and chemokine genes, including IL6, CXCL1, LTB, CXCL5, CXCL8, CSF2, and TGFβ2, were downregulated by WCE treatment compared to the control (Fig. 3a). The Ingenuity Pathway Analysis (IPA) of RNA-Seq data suggested that NFκB may be the common upstream regulator of these cytokines (Fig. 3b). Among these differentially expressed genes, pro-inflammatory cytokines, such as IL6, CXCL1, CXCL5, CXCL8, and CSF2 were further validated as significantly downregulated in WCE-treated PC-3 and DU145 tumor tissues (Fig. 3c). It is well-known that the inflammatory chemokines are regulated by the NFκB pathway. The WCE effect on the phosphorylation-mediated proteasome degradation of IκB was dose-dependent as analyzed by immunoblotting assays, which repressed NFκB in PC-3 and DU145 cells following WCE treatment (Fig. 3d). To validate this hypothesis, we then modulated the NFκB pathway by expression of either constitutively active or dominant negative mutants of IKKβ or IKKα in PC-3 cells. As expected, IKKα/β significantly controlled the mRNA level of these cytokines (Fig. 3e). IHC staining in tumor tissues demonstrated lower levels of expression of IL6, CXCL1, CXCL8 and IKK-mediated phosphorylation of p65 (S536) in WCE-treated tumors than control tumors, indicating the *in vivo* IKK activity was also suppressed by WCE (Fig. 3f). Plasma levels of tumor-derived human IL6 and CXCL1 were indeed ablated in WCE-treated mice compared to vehicle-treated mice (Fig. 3g).

### WCE modulates the tumor microenvironment by inhibiting myeloid cell infiltration and activity

**Figure 4:**
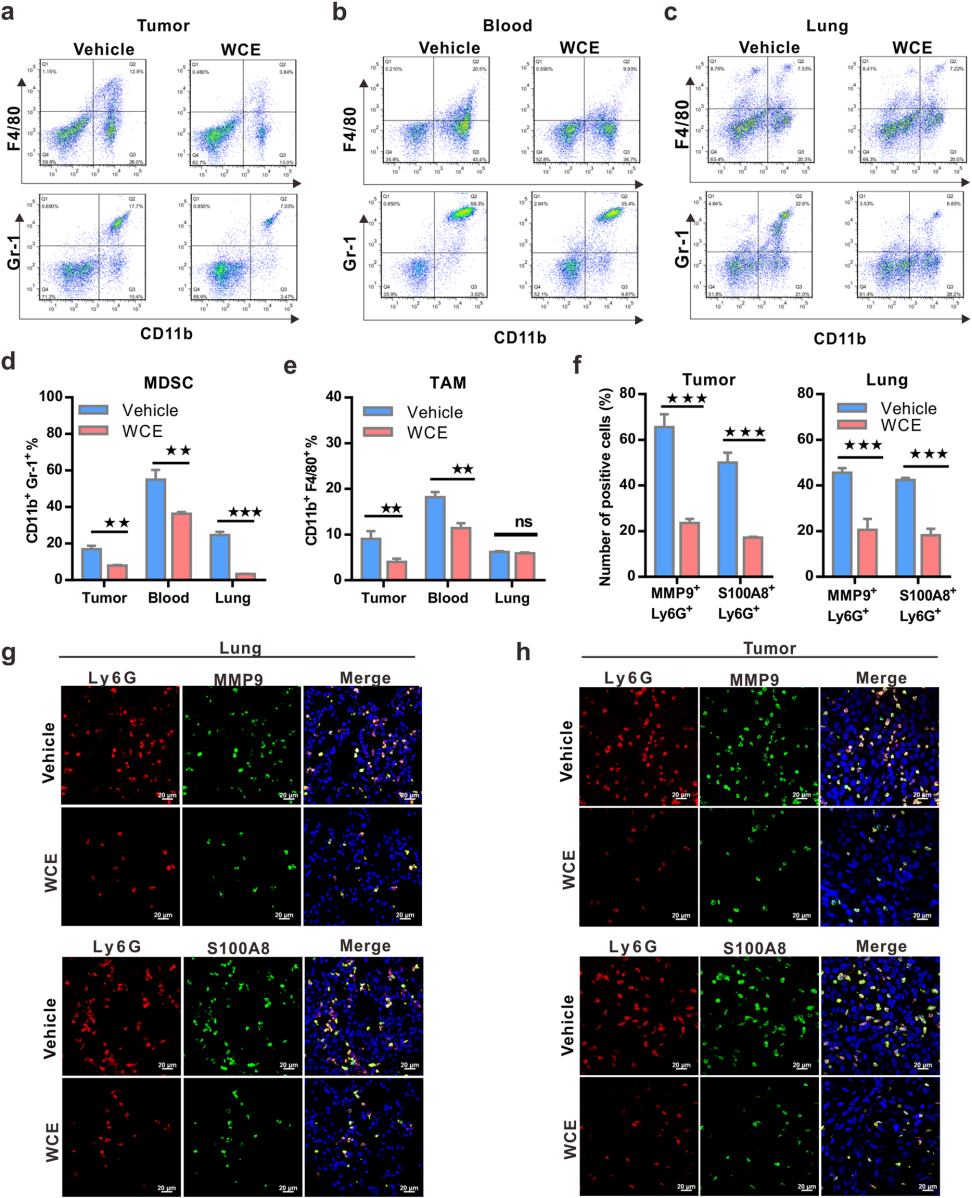
Effect of WCE on the mobilization and recruitment of myeloid cells in a nude mouse bearing PC-3 tumors. (**a**–**c**). The percentage of Gr-1^+^/CD11b^+^ myeloid cells and F4/80^+^/CD11b^+^ tumor-associated macrophages in primary tumors (**a**), peripheral blood (**b**) and lung (**c**) in tumor-bearing mice treated with WCE or vehicle as identified by FACS. (**d**,**e**) The percentage of MDSCs (**d**) and TAMs (**d**) in different tissues are shown as means ± SE (n = 3). (**f**) Fractions of Ly6G^+^/MMP9^+^ and Ly6G^+^/S100A8^+^ cells in primary PC-3 tumors (left) and in the lung (right) were calculated (n = 5). (**g**,**h**) The representative image of Ly6G^+^/ MMP9^+^ cells (**g**) and Ly6G^+^/S100A8^+^ cells (**h**) in primary PC-3 tumors and lungs were detected by immunofluorescent staining. Bar, 20 μm. All values are represented as means ± SEM. *P ≤ 0.05; **P ≤ 0.01; ***P ≤ 0.001 (t-test, two-tail).

**Figure 5:**
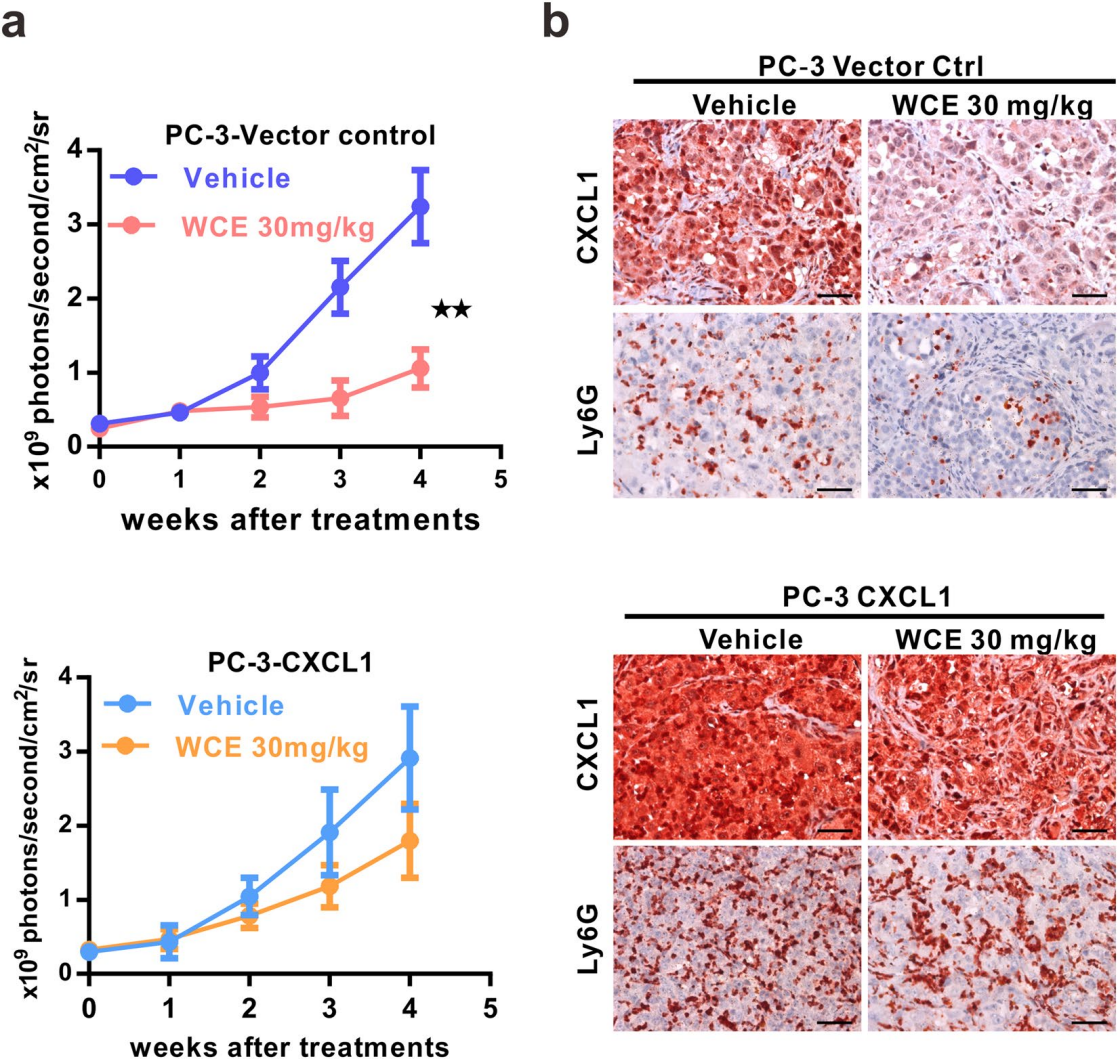
Effect of ectopic CXCL1 expression in PC-3 cells on WCE-mediated responses. (**a**) The tumor growth of PC-3 cells transfected with control vector or CXCL1 expression vector (PC3-CXCL1) was determined using the respective longitudinal BLI intensity. WCE or vehicle control was administered orally, daily for 4 weeks. (**b**) IHC analysis for the expression of CXCL1 and Ly6G in the orthotopic tumors of PC-3 vector control and PC-3-CXCL1 after treatment. Scale bar, 50 μm. Data are expressed as means ± SEM. **P < 0.01 (t-test, two-tail).

Since WCE affected the tumor-induced splenomegaly and cytokine response so profoundly, we postulated that WCE may mediate cancer development through modulating the tumor microenvironment. Therefore, we used FACS to analyze the population of tumor-associated macrophages (TAMs, F4/80^+^/CD11b^+^) and myeloid-derived suppressor cells (MDSCs, Gr1^+^/CD11b^+^) in the tumor, peripheral blood and lung tissues whose abundance might be changed upon WCE treatment in the PC-3 xenograft model. WCE treatment significantly decreased MDSCs and TAMs in primary tumors and peripheral blood, but this effect was limited to MDSCs in the lung tissue (Fig. 4a–e). The decreased populations of MDSCs and TAMs were also shown in WCE-treated DU145 tumor tissues (Supporting Information Fig. S1g). MDSCs are noted to secrete MMP9 to facilitate tumor invasion and metastasis and S100A8/9 to promote survival of cancer cells^19^. Indeed, the numbers of MMP9^+^ Ly6G^+^cells and S100A8^+^ Ly6G^+^ cells in the primary tumor and in the lung were significantly decreased by WCE treatment (Fig. 4f–h). On the other hand, the rescue of CXCL1 expression in the PC-3 tumors blunted the WCE-mediated immune modulation by increasing Ly6G^+^ MDSCs infiltration thus reversing the detrimental effect of WCE on the tumor growth (Fig. 5a and b). In conclusion, WCE effectively diminished the population of MDSCs in the tumor, lung, and blood circulation by targeting NFκB-mediated cytokines. These observations suggested that WCE may block the mobilization of MDSCs induced by PCa cells and the ensuing cancer metastasis.

**Figure 6:**
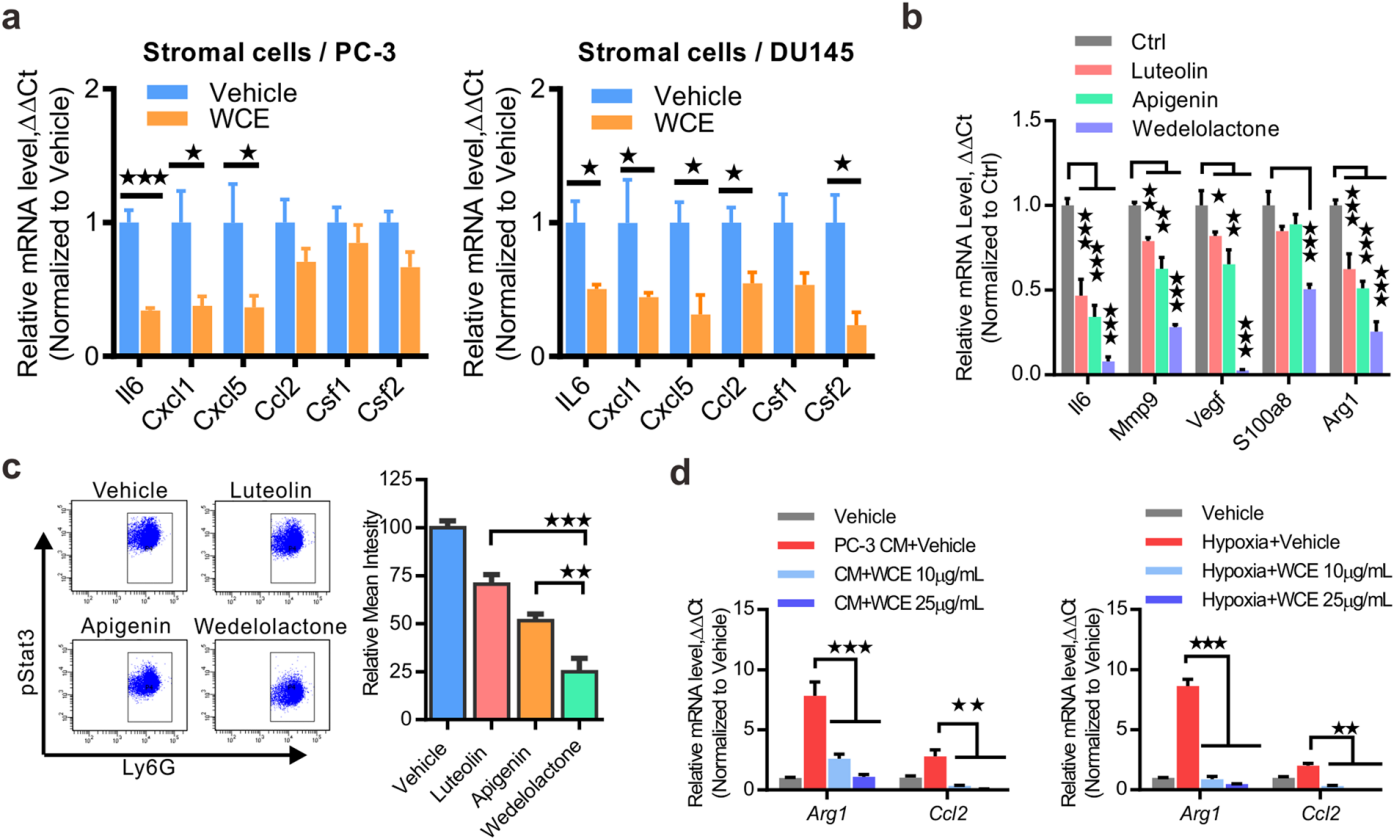
WCE modulates myeloid cell activity. (**a**) The cytokine/chemokine levels of stromal cells in PC-3 and DU145 tumor tissues were analyzed using mouse-specific primers and qRT-PCR. (**b**) Tumor-derived MDSCs were treated with the indicated compound (10 μmol/L) for 6 h, and proinflammatory genes were analyzed by qRT-PCR. (**c**) Phosphorylated Stat3 was determined in *ex vivo* culture of PC-3 tumor-derived MDSCs by FACS following indicated treatment at 10 μmol/L for 6 h. Right, quantitative mean values of triplicate. (**d**) M1 markers, arginase 1(Arg1) and Ccl2, were analyzed in qRT-PCR under indicated treatments of PC-3 CM or hypoxia (1% O_2_) plus WCE treatment or vehicle for 12 h. Data are expressed as means ± SEM. **P < 0.01, ***P < 0.001 by unpaired Student’s t-test.

To understand whether WCE might have a systematic effect on host cells, the host cell gene transcription of *Il6*, *Cxcl*1, *Cxcl5*, *Ccl2*, *Csf1*, and *Csf2* were analyzed using mouse-specific primers. The mRNA levels of these cytokines/chemokines exhibited a significant decrease in WCE-treated PC-3 and DU145 tumor samples (Fig. 6a). Further, the effects of individual active compounds were dissected using *ex vivo* culture of MDSCs enriched by the CD11b^+^/Ly6G^+^ population from PC-3 tumors and treatment with apigenin, luteolin, or wedelolactone for 6 h. Among the three major active compounds, wedelolactone had the most profound effect on suppression of inflammation-regulated genes, i.e., *Il6*, *Mmp9*, *Vegf-A*, *S100a8* and *Arginase 1* (*Arg1*) (Fig. 6b). Activation of STAT3 is a central immune checkpoint regulator in MDSC and regulates the expression of these genes. Following the *ex vivo* treatment of tumor-infiltrated MDSCs with individual active compounds, the intensity of pStat3 in CD11b^+^/Ly6G^+^ MDSCs was also decreased, most profoundly by wedelolactone followed by apigenin and luteolin over the vehicle control (Fig. 6c). The ability of WCE to suppress cytokine expression was also observed in bone marrow-derived macrophages (BMDMs). BMDMs stimulated with PC-3 cell conditioned medium (PC-3CM) or hypoxia exhibited an M2-like phenotype by upregulating the *Arg1* and *Ccl2*, which were completely inhibited by *ex vivo* treatment with WCE in a dose-dependent manner (Fig. 6d). In conclusion, tumor-induced inflammation was tightly restrained by WCE treatment in both PCa cells and myeloid cells.

### WCE augments the docetaxel chemotherapy while reducing toxic side effect in PCa

**Figure 7:**
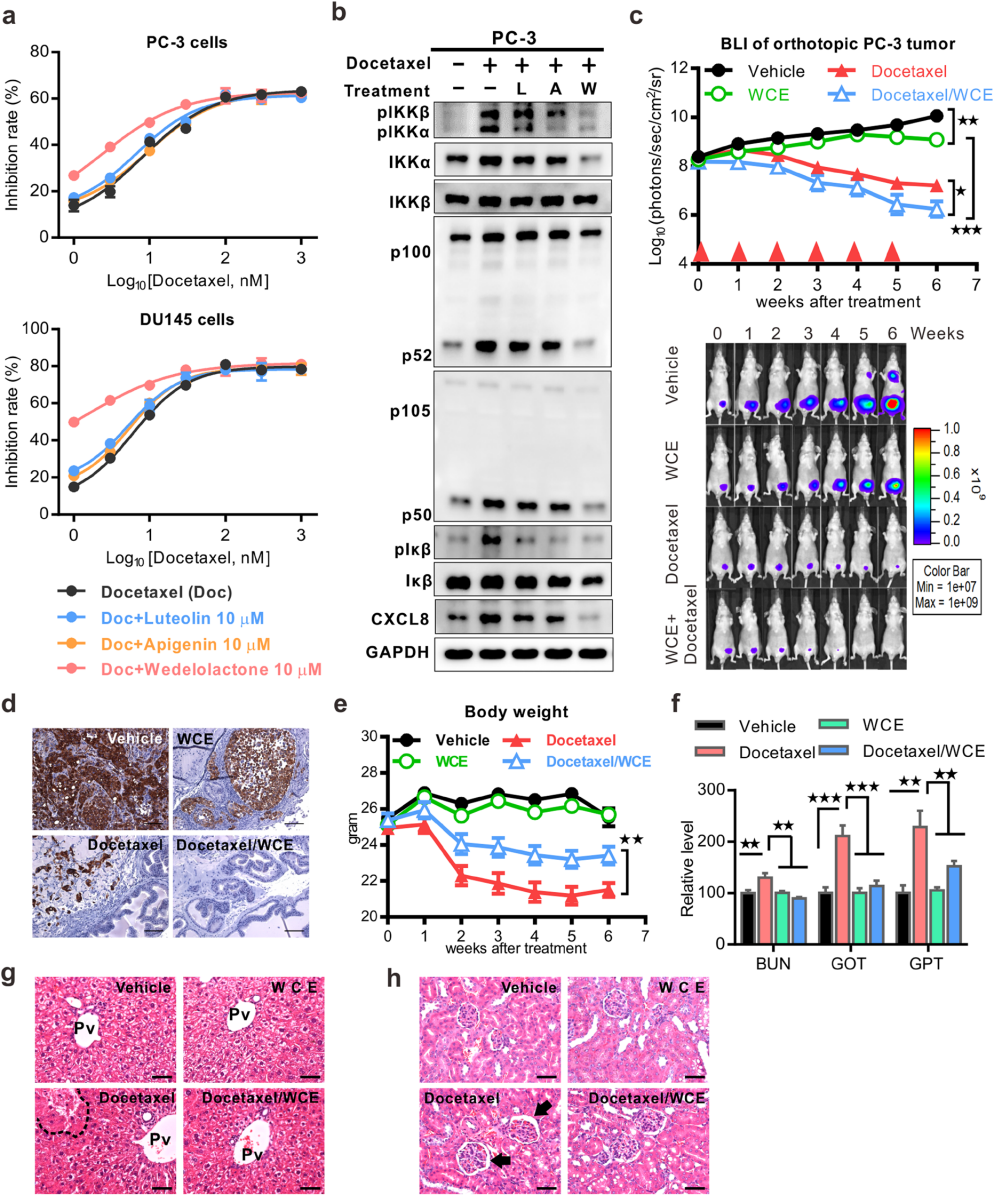
Effects of a combination of WCE with docetaxel on hormone-refractory prostate cancer. (**a**) Dose-response curves of PC-3 and DU145 cells following treatment with different concentrations of docetaxel alone or plus 10 μmol/L of luteolin, apigenin or wedelolactone. (**b**) WCE-mediated suppression effect on docetaxel (30 nM)-induced IKK phosphorylation and its downstream molecules were analyzed using western blot. *L*, luteolin 10 μmol/L. *A*, apigenin 10 μmol/L. *W*, wedelolactone 10 μmol/L. (**c**) Tumor growth curves were determined by measuring BLI intensity weekly following the indicated treatments of the vehicle, 30 mg/kg WCE, 10 mg/kg docetaxel or a combination. Bottom, longitudinal BLI images of representative mice in each group. (**d**) Cytokeratin-18 staining to detect PC-3 cells in primary tumors following indicated treatments. Scale bar, 50 μm. (**e**) The longitudinal mouse body weight in each group was plotted against time after treatment. (**f**) The systemic toxicity of different treatment was analyzed by measuring BUN (blood urea nitrogen), GOT (glutamic-pyruvic transaminase), and GPT (glutamic-oaa transaminase) in the plasma. (**g**,**h**) Pathological analyses of the liver (**g**) and kidney (**h**) tissues were examined by H&E staining. Pv, hepatic portal vein. The area of necrosis is enclosed by the dotted line. Arrow, necrotic tissue. Arrow, mesangial expansion. Scale bar, 50 μm. Data are expressed as means ± SEM. *P < 0.05, **P < 0.01, ***P < 0.001 by unpaired Student’s t-test.

Docetaxel is the first-line therapy for metastatic CRPC (mCRPC), which is an anti-mitotic agent and is primarily used to treat metastases in other neoplasms, including breast, gastrointestinal, head and neck, and non-small cell lung cancers. Clinical studies showed that patients receiving chemotherapy have increased NFκB activity and cytokine expression, such as CXCL8, and a high level of CXCL8 compromises the docetaxel-mediated apoptosis of tumor cells^14,15^. Theoretically, earlier treatment at the clinical stages of a locally advanced tumor or micrometastasis has a better chance of lowering the recurrence rate and eradicating small lesions of disseminated cells that may have developed the clonal diversity inclined to acquire resistance to docetaxel therapy. Docetaxel treatment in PCa cell lines induced the activation of IKKα and IKKβ that activated the canonical and alternative NFκB signaling pathways (Supporting Information Fig. S2a). WCE suppressed the docetaxel-induced activation of IKKα/β and also downstream IL6, CXCL8 and CXCL1 expression, which may counteract the therapeutic resistance to docetaxel (Supporting Information Fig. S2b and c). To the end, we analyzed the effect of docetaxel on the cell growth of PC-3 and DU145 cells and the impact of each active compound on the effect of docetaxel, including luteolin, apigenin, or wedelolactone. Among them, only wedelolactone significantly enhanced the cytotoxic effect of docetaxel in PC-3 and DU145 cells (Fig. 7a). Congruently, the inhibitory effects of wedelolactone were observed in the phosphorylation of IKKα and IKKβ, and also the downstream canonical and alternative NFκB signaling pathways by immunoblotting analyses (Fig. 7b). *In vivo*, monotherapy with either WCE or docetaxel reduced the tumor growth, and when used in combination the therapeutic effect was further intensified and eradicated the orthotopic PCa tumors (Fig. 7c and Supporting Information Fig. S2d). As a result, the BLI signal in all mice dropped below the detection limit (n = 10) at 7 weeks following the combination therapy; and pathological examination confirmed that no tumor cells were found in the prostate of the combination group, whereas some residual tumor cells were observed in that of monotherapy groups (docetaxel or wedelolactone) (Fig. 7d). Of note, the WCE and docetaxel combination not only had a more potent therapeutic effect than docetaxel but also recovered the body weight loss (Fig. 7e and Supporting Information Fig. S2e). Furthermore, biochemical analysis of kidney and liver injuries as determined by the levels of the BUN, GOT and GPT in the peripheral blood suggested that WCE lowered the docetaxel-mediated tissue damage in the liver and kidney (Fig. 7f). The histopathology specimens obtained from docetaxel-treated mice exhibited hepatocyte necrosis compared to those obtained from control and WCE groups (Fig. 7g). Specimens from the WCE-docetaxel combination group were obviously devoid of hepatocyte necrosis (Fig. 7g). Moreover, the group treated with docetaxel alone developed obvious mesangial expansion with interstitial hemorrhage, a morphologic characteristic of renal damage (Fig. 7h). The mesangial expansion and interstitial hemorrhage mediated by docetaxel were absent in the WCE-docetaxel combination group where the structures of the glomeruli were similar to the control (Fig. 7h). These results suggest that WCE was able to protect docetaxel-mediated tissue damage in tumor-bearing mice, at least in the liver and kidney. In conclusion, WCE enhanced the therapeutic effect of docetaxel, prevented metastatic progression, and also protected against docetaxel-induced tissue damage and chemotherapeutic resistance.

## Discussion

Herbal medicine usually reveals the issues for toxic effects and *in vivo* efficacy. *In vivo* flavonoids are rapidly conjugated with glucuronide and sulfate by liver metabolism for excretion. However, our unpublished data showed that 12 hours following a single oral dose of 100 mg/kg WCE by gavage in mice, wedelolactone, luteolin, and apigenin were still present at low but detectable levels specifically in prostate and liver, not kidney spleen, and others, indicating three active compounds selectively distributed in prostate and therefore the herbal compounds do not accumulate in most tissues to cause potential side effect. Also, quantified WCE must meet our criteria for the chemical content of >70% of luteolin, apigenin, and wedelolactone quantified by triple-quadrupole mass-spectrometry, which reduces the content of unknown components for any unpredictable adverse effect^18^. On the other hand, luteolin, apigenin, and wedelolactone are naturally occurring flavonoids that are abundantly and widely in vegetables, fruits, and medical herbs; the toxicity evaluation of luteolin, apigenin, and wedelolactone have been examined in rat model showing no significant adverse effects on kidney and liver and even exert a protective effect on the kidney and liver tissues from damages induced by carbon tetrachloride and chemotherapy, respectively^20–22^. Furthermore, since the synergistic effect of WCE by targeting different survival signaling pathways, such as AR and IKKα/β, WCE may start to exhibit its beneficial effect efficiently at a low dose *in vivo* without the need for a high dosage and reduce the risk of adverse effect.

Although liver metabolism leads rapid elimination of flavonoids especially wedelolactone, all the previous studies were analyzing the scenarios of a pure single compound or preparations with no medicinal effects^23,24^. In contrast to the single use of pure flavonoids, *Wedelia chinensis* extract benefits from multiple components due to the pharmacokinetic synergy which modulates the metabolism to increase the *in vivo* half-life of three active compounds and the pharmacodynamic synergism on suppressing tumor growth, therefore lowers the amounts of active compounds in WCE required for *in vivo* anti-cancer activity^17,18^. Furthermore, the convergence of previous studies and our finding in this work indicated that WCE through its three active compounds and synergisms exert not only suppressing cancer cell growth but also immunomodulation effect on cytokine expression from cancer cells and STAT3 activity in tumor-elicited myeloid cells. Altogether, the combination of herbal compounds includes pharmacokinetic and pharmacodynamic synergisms to provide significant therapeutic effects *in vivo*.

The anti-cancer activity of *Wedelia chinensis* and its ingredients have been studied and evaluated in several *in vitro* and *in vivo* cancer model. For example, the essential oil extracted from *Wedelia chinensis* using hydro-distillation contained carvacrol and trans-caryophyllene which had various antioxidant properties *in vitro* and *in vivo* ^25^. A previous study indicated the ethyl acetate partition-enriched unknown compounds inhibit nasopharyngeal cancer CNE-1 cells by inducing Chk1-mediated G2/M arrest and suppressing MYC transcription^26^. In agreement with our early study, we found that apigenin, luteolin, and wedelolactone were enriched in the ethyl acetate fraction which exerts the apoptosis effect on AR-positive LNCaP and 22Rv1 and results in the G2/M arrest of PC-3 cells^16^. Additionally, the natural bioflavonoids of apigenin, luteolin, and wedelolactone have been shown to possess potent therapeutic properties in prostate and breast cancers. Wedelolactone has been reported downregulates the expression of MYC mRNA in prostate cancer cells^27^. Also, wedelolactone was reported to induce caspase-dependent apoptosis in prostate cancer cell line LNCaP by downregulating PKCε^28^. Moreover, inhibition of IKKα by wedelolactone decreases the nuclear localization of AR and suppresses AR signaling^29^. In breast cancer model, wedelolactone suppresses growth and induces apoptosis of MDA-MB-231 by inhibiting the activity of DNA topoisomerase IIα^30^. On the other hand, luteolin and apigenin are found widely in many type plants including fruit, vegetables, and traditional medicinal herbs and exert their anticancer activity by inhibiting the AR expression, VEGF-mediated angiogenesis and IGF1R signaling in prostate cancer^31–34^. Additionally, luteolin treatment can induce E-cadherin expression, decrease *in vitro* invasion activity, and suppress lung metastasis in s.c. PC-3 tumor model at 5 mg/kg thrice a week through intraperitoneal injection; oral administration of apigenin with 50 μg/mouse/day increases E-cadherin, inhibit nuclear translocation of β-catenin, and prolong survival of PCa transgenic mice TRAMP^35,36^. Here, we further identified that therapeutic targets of WCE by suppressing the activation of IKKα/β signaling and STAT3 activity *in vivo*, therefore exhibiting immunomodulation function and beneficial effect on suppressing docetaxel-induced cytokines expression.

The NFκB pathway is regarded as an emerging cancer drug target because NFκB transcription factors are crucial regulators associated with numerous hallmarks of cancer, including inflammatory (IL6, CXCL1, CXCL5, CXCL8, and MCP1), proliferation (MYC, CCND1, CCNE2, and CDK2), metastatic (MMP2/9), and angiogenic (VEGF)^37^. Increased expression of CXCL1, CXCL5 or CXCL8 has been shown in advanced PCa, and the CXCL1, 5 and 8 chemokines share the same receptor CXCR2^38–40^. In addition, CXCL1 activates NFκB signaling to promote PC-3 cell survival, migration, and invasion^41,42^. CXCL8 expression contributes to androgen-independent growth, metastasis, and chemoresistance by upregulating AR, MMP9, and NFκB, respectively^43^. Androgen-deprivation therapy (ADT) or hormonal therapy has been shown to induce tissue inflammation in PCa. A lot of immune B cells and macrophages are attracted into the castrated tumor and then release enough cytokines to inhibit apoptosis and promote the growth of tumor cells^44,45^. In a preclinical tumor model, ADT also enhances the expression of macrophage-recruiting chemokine CSF1 and M2-promoting cytokines, such as IL13 and IL10 in PCa cells, resulting in more macrophage infiltration and expression of MMP9 and VEGF^46^. Therefore, long-term inflammation is postulated to contribute to the failure of ADT and perhaps the development of CRPC. Consequently, inflammation and immune cell infiltration highly affect the treatment outcome of PCa. After relapse from ADT, there are only limited therapeutic options available to patients, rendering CRPC a significant public health burden. In this study, we demonstrated that WCE treatment significantly inhibited the phosphorylation of IKKα/β, abolished NFκB-mediated expression of cytokines and chemokines in the tumor, and also limited MDSCs infiltration into the tumor and distant organs. Furthermore, we showed that wedelolactone has a more potent effect than luteolin and apigenin, simultaneously inhibiting canonical and non-canonical NFκB pathways. Of note, our previous study also indicated that wedelolactone plays a dominant role in determining the activity of different WCE batches and qualifying the WCE potency of *in vivo* anti-tumor growth and *in vitro* anti-AR activity^18^.

Both IKKα and IKKβ can directly phosphorylate IκB, resulting in its ubiquitination and degradation by 26 S proteasome to release NFκB for gene regulation^47^. Therefore, targeting both IKKα and IKKβ effectively impairs the NFκB signaling. On the other hand, the non-canonical IKKα pathway plays a unique role in regulating cancer progression. In the alternative NFκB pathway, IKKα/α homodimer phosphorylates p100, resulting in limited degradation of p100 into p52 by the proteasome, followed by nuclear translocation of the RELB-p52 heterodimer^48^. In PCa models, the activation of IKKα by the receptor activator of NFκB ligand (RANKL) signaling through RANK promotes metastasis^49^. IKKα can cross-talk with AR to promote PCa cell survival^29^. Together these mechanisms contribute to the dominant role of wedelolactone in determining the anti-tumor activity of WCE.

Many cellular and molecular components of the immunosuppressed tumor microenvironment have been identified as potential targets for treating advanced PCa, including MDSCs, NFκB, and pro-inflammatory protein S100A8/9. In clinical PCa, patients with metastases have a significantly higher number of granulocytic MDSCs, not monocytic MDSCs in circulation compared to healthy subjects or patients with localized disease, which may serve as a potential target for PCa treatment^50^. MDSC-derived S100A8/A9 and MMP9 can pre-dispose the lung microenvironment to promote tumor metastasis^51^. On the other hand, STAT3 plays a critical role in the MDSC expansion by stimulating myelopoiesis and inhibiting myeloid-cell differentiation through up-regulation of S100A8/A9^52^. MDSCs promote tumor metastasis and angiogenesis via secreting VEGF and MMP9 which is also regulated by IL6-STAT3 activation^53^. In a preclinical PCa animal model, the CSF1R blockade was shown to restrict the local expansion of MDSCs, thus maintaining the immune surveillance against tumor growth^54^. S100A8/9, correlated with PCa progression, can regulate MDSC expansion and also trigger the NFκB pathway in cancer cells to promote proliferation and migration^55^. Tasquinimod is an oral quinoline-3-carboxamide derivative currently in phase III clinical studies, which can engage with S100A9 to block its binding to pro-inflammatory receptors such as RAGE and TLR4. In a phase II study of 201 men with mCRPC, tasquinimod treatment extended the median progression-free survival from 3.3 to 7.6 months^56^. Our study showed that the S100A8 expression and MDSCs infiltration were diminished by WCE and these effects were reversed by CXCL1. This result is sustained by another study in which CXCL1/2 knockdown decreased the CD11b^+^Ly6G^+^ granulocytic population with high-level CXCR2 instead of monocytic MDSCs and suppressed breast cancer progression^57^.

As an adjuvant therapy, WCE enhanced the efficacy of docetaxel treatment in an animal model by inhibiting NFκB and downstream CXCL8. Docetaxel induces NFκB activation and upregulation of CXCL8, which can compensate docetaxel-induced cell death. Therefore, a combination of WCE and docetaxel chemotherapy augmented efficacy but lowered toxicity. Moreover, WCE significantly downregulated STAT3/S100A8 signaling, limited the expansion of MDSCs, and targeted the release of downstream chemokines from cancer cells, which prevented the tumor microenvironment entering a vicious cycle. In conclusion, WCE curbed chemokine expression, modulated the tumor microenvironment, and enhanced docetaxel chemotherapy while reducing adverse side-effects in mouse models of hormone-refectory PCa, suggesting that the standardized preparation of *W. chinensis* extract may improve the therapeutic outcome either as an add-on to docetaxel treatment or as a monotherapy for CRPC. WCE is worth further investigation in clinical trials as either a neoadjuvant or adjuvant regimen.

## Methods

### Chemical reagents and antibodies

Commercial antibodies against IL6, CXCL8, p65 (GeneTex International Corp, Hsinchu City, Taiwan), p105/p50, p100/52 (EMD Millipore, Billerica, MA), pIKKα/β, pIκB (Cell Signaling Technology, Danvers, MA), IκB (Santa Cruz, Dallas, Texas) and CD31, phosphorylated p65, IKKβ, IKKα and pSTAT3 (Abcam, Cambridge, MA) were used. Chemokine ELISA kits were from R&D systems (Minneapolis, MN). Docetaxel was from Enzo Life Sciences (Farmingdale, NY). Fluorescent dye-conjugated antibodies used in the flow cytometry analysis were all from Biolegend (San Diego, CA).

### Cell lines

LNCaP, 22Rv1, PC-3, and DU145 cell lines were obtained from American Type Culture Collection (ATCC, Manassas, VA) between 2002 and 2006 and authenticated by comparing in ATCC database of short tandem repeat (STR) loci within 6 months of the last experiment. The STR DNA profile analysis was performed by using the Promega GenePrint system and analyzed by ABI PRISM 3730 Genetic Analyzer and GeneMapper software v.3.7. All cell lines were routinely cultured in RPMI-1640 supplemented with 2 mM glutamine, 1 mM sodium pyruvate, and 10% fetal bovine serum.

### Preparation of *Wedelia chinensis* extract

The whole fresh plants were air-dried, ground and extracted by immersion with ethanol. After condensing, the ethanolic extract was acid-hydrolyzed with HCl at pH 2.0, 80 °C for 30 min to increase the aglycone flavonoid content, then neutralized with NaOH and applied to a flash LC system using a C18 column (SNAP 400 KP-C18-HS Column, Biotage, Uppsala, Sweden) to separate the extract into fractions. To assure consistent WCE quality, the chemical profiles of all WCE lots were analyzed by HPLC-CAD, quantified by LC-MS triple quadruple for luteolin, apigenin, and wedelolactone content, and analyzed by PSA reporter assay for the AR-inhibitory activity of WCE. The WCE was dried as a powder and stored in a freezer at −80 °C for later use.

### Cell growth assay and qRT-PCR

For cell growth assay, 5000–10000 cells were seeded in 96-well plates overnight and treated with different concentrations of inhibitor for 2 days. The readouts of cell number are measured by CyQUANT Direct Cell Proliferation Assay (Invitrogen). For measurement of WCE-mediated gene expression, cells were seeded in 6-well plates and treated with WCE, docetaxel, or a combination of both for 24 h. Quantitative polymerase chain reaction (qPCR) analysis was performed using a ThermoFisher scientific SYBR Master Mix and an ABI real-time PCR system.

### *Ex vivo* analysis of WCE effect on tumor-associated myeloid-derived suppressor cells

Mice were orthotopically inoculated with 2 × 10^5^ PC-3 cells, and the tumors were allowed to grow for 6 weeks. The tumor tissues were digested in collagenase for 1 h, and MDSCs were isolated by FACS using anti-CD11b and Ly6G antibodies. Cells were cultured in RPMI-1640 medium containing HL-1 supplement, β-mercaptoethanol, and glutamine, and treated with apigenin, luteolin or wedelolactone for 6 h to analyze pSTAT3 level and downstream gene responses in flow cytometry and qRT-PCR, respectively.

### Animal study

Athymic nude mice (6 weeks old) were obtained from the National Laboratory Animal Center (Taiwan), and all animal work was conducted in accordance with a protocol approved by the Institutional Animal Care and Use Committee, Academia Sinica. For *in vivo* measurement of tumor progression, cells were stably transfected with firefly luciferase EF1α/eIF4g-luc2^58^. For orthotopic implantation, male mice were anesthetized with 2.5% isoflurane, and 2 × 10^5^ cancer cells in 20 μL of DPBS were injected into the anterior prostate. After 1 week, mice were treated daily with WCE (30 mg/kg) through gavage, weekly with docetaxel (10 mg/kg) through tail vein injection, or a combination of both treatments for the indicated times. To determine tumor size, bioluminescence imaging (BLI) intensity of tumors was monitored weekly. At end-point mice were sacrificed by CO_2_ overdose and individual tumors were weighed, and then fixed in 10% formalin for pathology analysis, or snap frozen in liquid nitrogen for biochemical analyses. For *ex vivo* BLI, tissues were excised, immersed in DPBS and imaged for 10 seconds.

### Immunochemistry staining

The formalin-fixed, paraffin-embedded tissue sections were deparaffinized and hydrated in a series of graded alcohol to water. After antigen retrieval and specific antibody incubation, Histofine polymer detection systems (Nichirei Biosciences, Tokyo, Japan) were used to detect the primary antibodies. Following substrate development, the slides were counterstained and mounted for light microscopy analysis. Pathological images were captured with a Zeiss AxioCam HRc camera attached to a Zeiss AxioImager.Z1 microscope (Munich, Germany). For determination of S100A8/Ly6G and MMP9/Ly6G positive cells, the five serial tissue sections in the lower lobe of the left lung (n = 3) were stained by immunofluorescence assay; images were acquired using a Zeiss LSM 510 confocal microscope and quantified using Zeiss AxioVision Rel.4.8 software.

### Statistics

Statistical analysis was performed using GraphPad Prism 6 software. Unpaired Student’s t-test (two-tailed) was performed as indicated. A P value of less than 0.05 was considered statistically significant. All values presented in the study are expressed as means with standard error (SEM).

### Data availability

The datasets analyzed during the current study are available in Gene Expression Omnibus (GEO) repository, GSE99820.

## Electronic supplementary material

Supplementary data
