# Supplementary material for: A standardized herbal extract mitigates tumor inflammation and augments chemotherapy effect of docetaxel in prostate cancer
Source: Sci Rep. 2017 Nov 15;7:15624. doi: 10.1038/s41598-017-15934-0 (PMC5688072; doi:10.1038/s41598-017-15934-0)
Supplement: Supplementary file 1 — Supplementary data [file 41598_2017_15934_MOESM1_ESM.pdf]

Supplementary Information

A standardized herbal extract mitigates tumor inflammation and augments chemotherapy effect of docetaxel in prostate cancer

Chin-Hsien Tsai[1], Sheue-Fen Tzeng[1,2], Shih-Chuan Hsieh[1], Yu-Chih Yang[1], Yi-Wen Hsiao[3], Mong-Hsun Tsai[1,3,4], and Pei-Wen Hsiao[1,2,*]

[1]Agricultural Biotechnology Research Center, Academia Sinica, Taipei, Taiwan;

[2]Graduate Institute of Life Sciences, National Defense Medical Center, Taipei, Taiwan;

[3]Bioinformatics and Biostatistics Core, Center of Genomic Medicine, National Taiwan University, Taipei, Taiwan

[4]Institute of Biotechnology, National Taiwan University, Taipei, Taiwan;

*Correspondence to Pei-Wen Hsiao, E-mail: pwhsiao@gate.sinica.edu.tw.

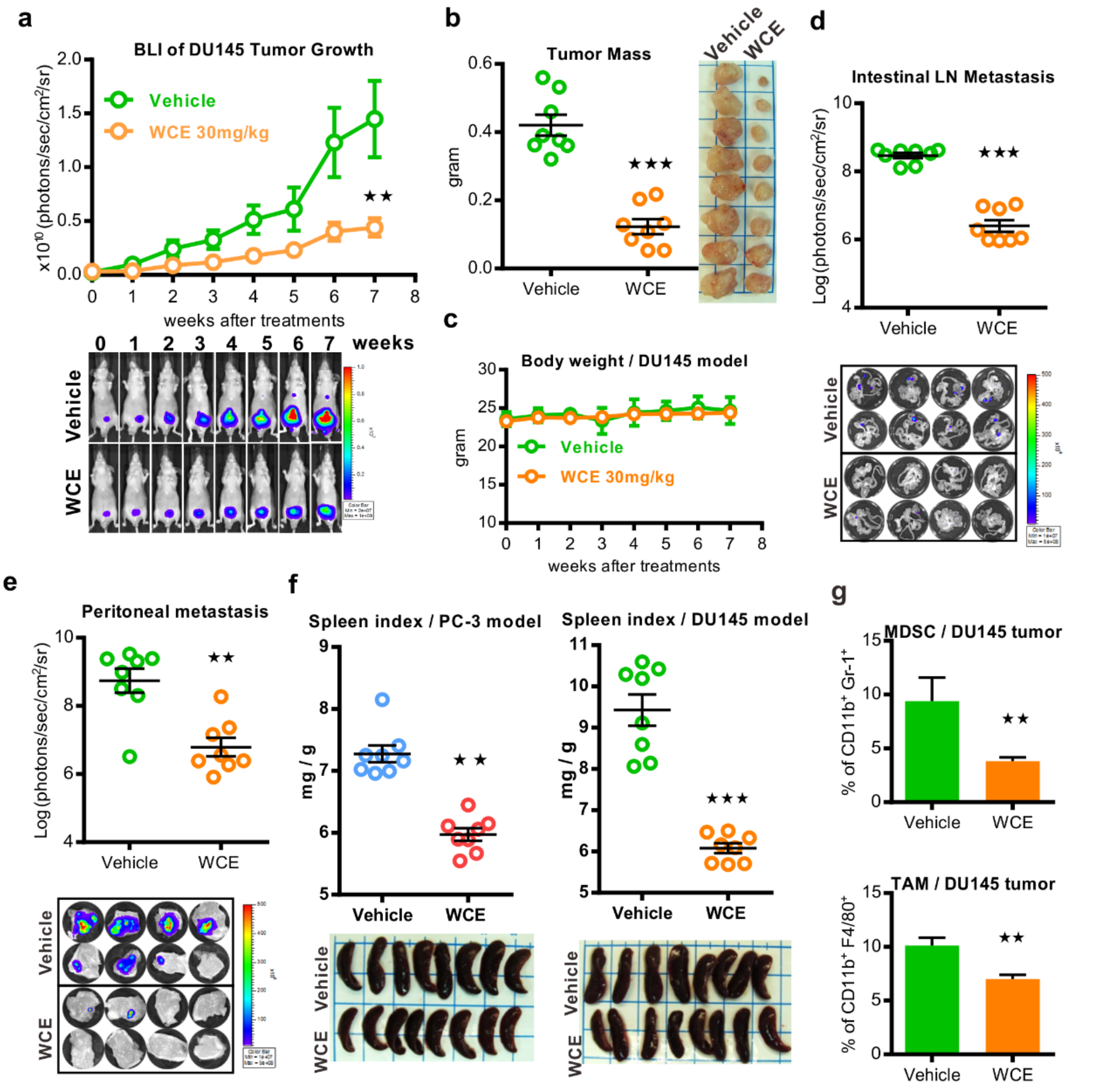

Figure S1. In vivo effects of oral WCE on orthotopic DU145 tumor model. (a) The longitudinal BLI of DU145-Luc2 prostate tumors in each group were measured weekly and are presented as a growth curve (n = 8). Bottom, the longitudinal images of BLI in representative mice. (b) The tumor weight following 8-week treatment is represented as a column scatter plot (n = 8). Right, image of representative primary tumors taken at endpoint. (c) Mouse body weight was recorded weekly. (d, e) Effect of WCE on tumor metastasis. BLI of intestinal lymph nodes (d) and intraperitoneal metastases (e) were examined at endpoint. Metastasis was quantified by BLI as photon flux per second and the results are represented as a column scatter plot. Horizontal lines, mean; bars, SE. (n = 8). (f) The spleen index (normalized against body weight) is presented in a bar graph and statistically compared with the control group. Left, spleen index and images in the PC-3 tumor model. Right, spleen index in the DU145 tumor model. (g) The populations of MDSCs and TAM in tumors following treatment for 7 weeks were analyzed using flow cytometry. Data are presented as mean ± SEM. **, $P \leq 0.01$, ***, $P \leq 0.001$. (t-test, two tail).

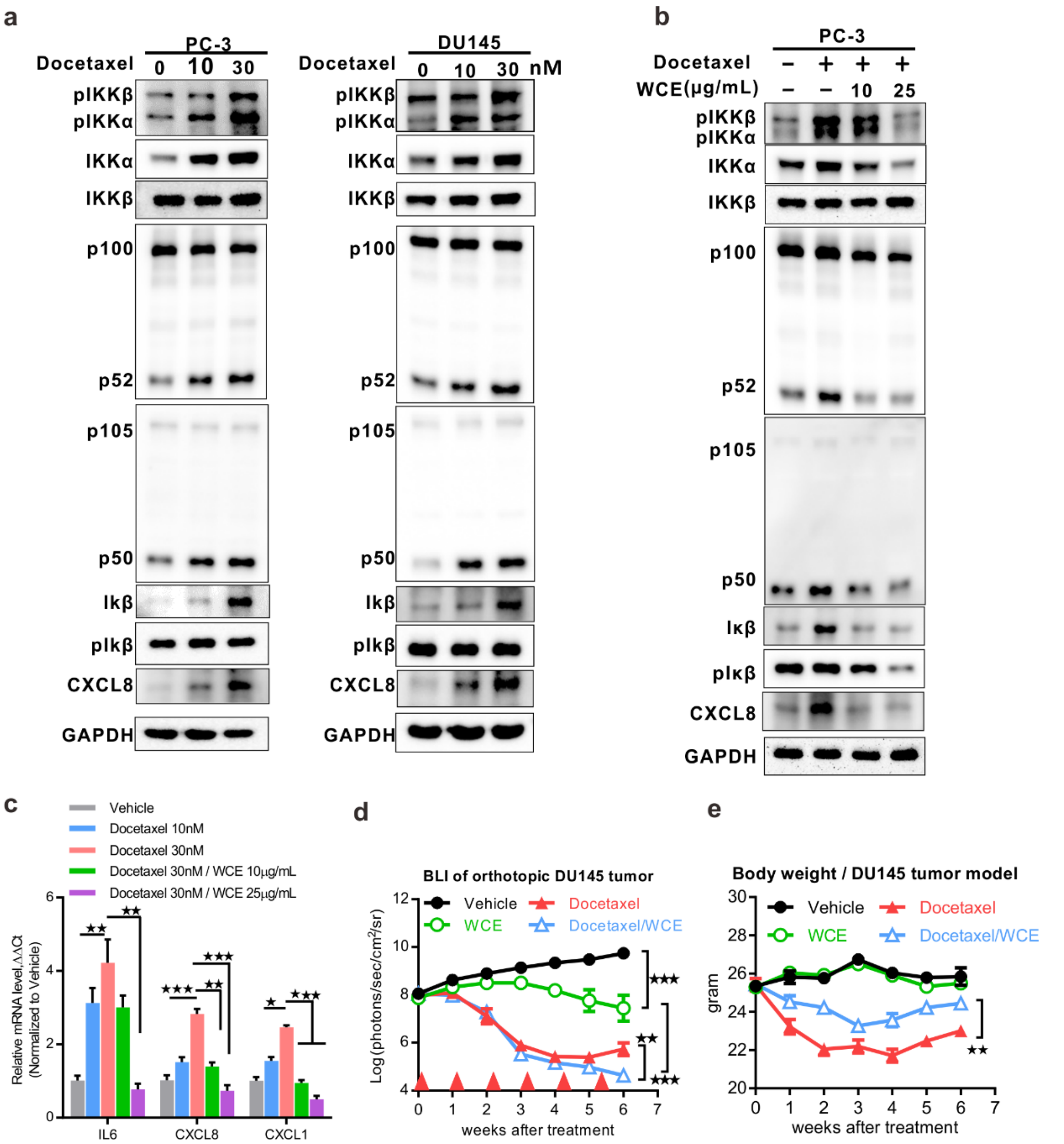

Figure S2. WCE suppressed docetaxel-mediated activation of IKK and augmented the therapeutic effect of docetaxel. (a) Docetaxel-induced IKKα/β activation and downstream CXCL8 were analyzed in PC-3 and DU145 cells using immunoblotting. (b) The effect of WCE on docetaxel (30 nM)-induced NFκB activity were determined by western blot. (c) WCE-mediated expression of proinflammatory cytokines in PC-3 was determined by RT-PCR following docetaxel stimulation. (d, e) The effect of the combination treatment was evaluated in the DU145 tumor model following treatment, vehicle, 30 mg/kg WCE, 10 mg/kg docetaxel or combination. Left, BLI of tumor growth curve are presented as mean ± SE. Right, the time course of mouse body weight in each group was plotted against time after treatment. Data are presented as mean ± SEM. **, P ≤ 0.01, ***, P ≤ 0.001. (t-test, two tail).
